# Evaluation of the Anti-Cancer Effects of KMU-11342 in In Vitro and Ex Vivo Models of Colorectal Cancer

**DOI:** 10.3390/ph19070985

**Published:** 2026-06-25

**Authors:** Jieun Jeon, Jeongin Jang, Chae Young Moon, Jinho Lee, Victor Sukbong Hong, Hyunju Kang, Jee Young Park, Na Hyeon Heo, Jong-Wook Park, Jae-Hyung Park, Jae-Ho Lee, Hye Won Lee, Sung Uk Bae, Hyunsu Lee, Shin Kim

**Affiliations:** 1 Department of Immunology, School of Medicine, Keimyung University, Daegu 42601, Republic of Korea; jje5726@gmail.com (J.J.); jeeyoung0610@hanmail.net (J.Y.P.); hnh9108@naver.com (N.H.H.); j303nih@dsmc.or.kr (J.-W.P.); 2 Department of Physiology, School of Medicine, Pusan National University, Yangsan-si 50612, Republic of Korea; jeongin122201@gmail.com; 3 Department of Food and Nutrition, Keimyung University, Daegu 42601, Republic of Korea; mm0917@naver.com; 4 Department of Chemistry, Keimyung University, Daegu 42601, Republic of Korea; jinho@kmu.ac.kr (J.L.); victorh@kmu.ac.kr (V.S.H.); 5 School of Food Science and Biotechnology, Kyungpook National University, Daegu 41566, Republic of Korea; hyunjukang@knu.ac.kr; 6 Department of Physiology, School of Medicine, Keimyung University, Daegu 42601, Republic of Korea; physiopark@kmu.ac.kr; 7 Department of Anatomy, School of Medicine, Keimyung University, Daegu 42601, Republic of Korea; anato82@dsmc.or.kr; 8 Institute of Medical Science, School of Medicine, Keimyung University, Daegu 42601, Republic of Korea; hwlee@dsmc.or.kr (H.W.L.); hispower@dsmc.or.kr (S.U.B.); 9 Institute for Cancer Research, School of Medicine, Keimyung University, Daegu 42601, Republic of Korea; 10 Department of Pathology, School of Medicine, Keimyung University and Dongsan Hospital, Daegu 42601, Republic of Korea; 11 Department of Surgery, School of Medicine, Keimyung University and Dongsan Hospital, Daegu 42601, Republic of Korea; 12 Research Institute for Convergence of Biomedical Science and Technology, Pusan National University Yangsan Hospital, Yangsan-si 50612, Republic of Korea; 13 Medical Research Institute, School of Medicine, Pusan National University, Yangsan-si 50612, Republic of Korea

**Keywords:** colorectal cancer, multi-protein kinase inhibitor, G2/M cell cycle arrest, apoptosis, GSK3β, CDK1, molecular docking, spheroid culture, patient-derived organoids

## Abstract

**Background/Objectives**: Colorectal cancer (CRC) remains one of the leading causes of cancer-related morbidity and mortality worldwide. Despite advances in treatment, outcomes for advanced CRC remain unsatisfactory due to uncontrolled proliferation, metastasis, and recurrence. This study investigated the anti-cancer effects of KMU-11342, an indolin-2-one-based multi-protein kinase inhibitor with previously reported anti-inflammatory properties, in human colorectal cancer models. **Methods**: The anti-cancer effects of KMU-11342 were evaluated in colorectal cancer cells and further investigated in three-dimensional (3D) spheroid and patient-derived organoid models. Cell proliferation, migration, apoptosis, and cell cycle progression were assessed. Kinase activity profiling and molecular docking analyses were performed to identify potential targets and characterize the underlying signaling pathways. **Results**: KMU-11342 significantly inhibited the proliferation and migration of CRC cells. It reduced CRC cell density by 58.9% and 83.3% at 0.5 and 1 μM, respectively. These effects were accompanied by G2/M cell cycle arrest and apoptotic cell death. In 3D models, spheroid formation was markedly reduced and stemness-related characteristics were diminished. Patient-derived CRC organoids also showed decreased viability, exhibiting 38.6% and 77.4% reductions at 1 and 2 μM, respectively. These effects were observed in a dose-dependent manner in both two-dimensional (2D) and 3D colorectal cancer models. Kinase activity profiling and molecular docking analyses identified glycogen synthase kinase 3 beta (GSK3β) and cyclin-dependent kinase 1 (CDK1) as potential mediators of the anti-cancer effects of KMU-11342 through the p53/nuclear factor kappa B (NF-κB) and FoxO1 signaling axes, respectively. **Conclusions**: KMU-11342 exhibits potent anti-tumor activity against CRC through suppressing proliferation, migration, and stemness in both 2D and 3D models, including patient-derived organoids. Its effects may be mediated, at least in part, through modulation of GSK3β and CDK1 via the p53/NF-κB and FoxO1 signaling pathways.

## 1. Introduction

Colorectal cancer (CRC) is the third most diagnosed cancer and second leading cause of cancer-related deaths worldwide, accounting for approximately 10% of all cancer cases and deaths [1]. Despite progress in screening programs and therapeutic approaches, including surgery, chemotherapy, and targeted therapy, treatment outcomes for advanced CRC remain suboptimal due to resistance to apoptosis, uncontrolled proliferation, and tumor recurrence [1]. Consequently, the development of novel therapeutic agents capable of overcoming these limitations remains a critical unmet need in CRC treatment.

Aberrant activation of kinase signaling pathways is a hallmark of CRC pathogenesis. Protein kinases play a pivotal role in the regulation of various cellular processes, including proliferation, survival, differentiation, and apoptosis, and their deregulation has been closely associated with tumor initiation, progression, and resistance to therapy. Among the most frequently implicated cascades are the PI3K/AKT, Wnt/β-catenin, NF-κB, and JAK/STAT pathways [2,3,4,5,6]. In particular, the PI3K/AKT and Wnt/β-catenin signaling pathways are frequently dysregulated in CRC and contribute to aggressive tumor behavior and reduced treatment efficacy [6,7]. Collectively, these findings position kinase inhibition as a rational and promising therapeutic strategy in CRC. Although single-target kinase inhibitors have shown therapeutic potential in cancer treatment, inhibition of a single target may be insufficient to effectively suppress tumor growth, as multiple signaling pathways are involved in CRC development and progression [2,8,9]. In this context, multi-target kinase inhibitors have attracted attention as a promising therapeutic strategy because they may enhance anti-cancer efficacy through simultaneous modulation of multiple signaling pathways involved in tumor progression [10,11].

Since the development of sunitinib, 2-indolinones have become a principal pharmacophoric building block in anticancer drug discovery. To this end, the indolin-2-one scaffold, present in the structures of several clinically used kinase inhibitors, has been widely investigated in kinase inhibitor and anticancer drug development. Structural modification of the indolin-2-one scaffold has enabled the development of compounds targeting diverse cancer-related kinases, including VEGFR, EGFR, PDGFR, FGFR, FLT3, CDKs, PI3K, and Aurora [11].

We previously developed KMU-11342, a novel multi-protein kinase inhibitor based on an indolin-2-one scaffold, and reported its anti-inflammatory activity [12]. Given the reported activity of indolin-2-one derivatives against diverse cancer-related kinase targets [11], we sought to investigate whether KMU-11342 could exert anti-cancer effects in CRC. In this study, we investigated the anti-cancer potential of KMU-11342 using CRC cells, 3D spheroid models, and patient-derived organoids.

## 2. Results

### 2.1. Kinase Activity Profiling of the Multi-Protein Kinase Inhibitor KMU-11342

**Figure 1:**
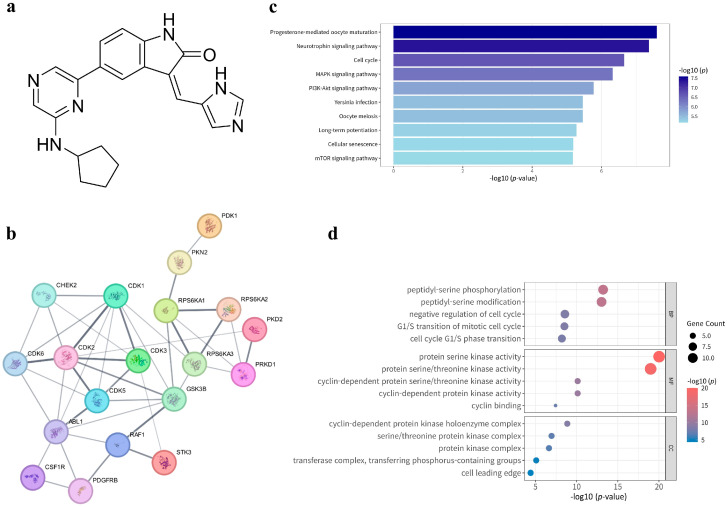
Pathway and network analysis of kinases exhibiting ≥80% activity inhibition following KMU-11342 treatment. (**a**) Chemical structure of KMU-11342, a multi-protein kinase inhibitor based on an indolin-2-one scaffold. (**b**) PPI network of the identified kinases. Nodes represent individual kinases, and edges represent protein–protein associations. Edge thickness indicates interaction confidence. Only interconnected kinases are displayed. (**c**) KEGG pathway enrichment analysis showing the top 10 enriched signaling pathways based on −log10 (*p*-value). (**d**) GO enrichment analysis across Biological Process, Molecular Function, and Cellular Component categories, showing the top 5 representative enriched terms in each category according to −log10 (*p*-value) and gene count.

KMU-11342, synthesized as previously reported (Figure 1a), was used in this study. Kinase profiling of KMU-11342 was performed using a panel of 67 cancer-related kinases at a concentration of 1 µM (Table 1). KMU-11342 exhibited the strongest inhibitory effects on GSK3β (−3%) and MST2 (−2%), followed by CDK1/Cyclin B (−1%), Abl (0%), CDK2/Cyclin A (0%), CDK5/p35 (1%), CDK2/Cyclin E (2%), and CDK3/Cyclin E (5%), indicating consistent suppression of several cyclin-dependent kinases (CDKs). Modest inhibition was observed in other cell cycle and survival-associated kinases, including CDK6/Cyclin D3 (5%), Rsk1 (5%), PKD2 (6%), PRK2 (8%), and c-RAF (10%). In contrast, KMU-11342 had minimal to no inhibitory effect (≥50% activity) on kinases such as PI3K (p110α/p85α), EGFR, Aurora-A, ATM, and ATR, suggesting target selectivity.

### 2.2. Pathway and Network Analysis of KMU-11342

Signaling pathways and biological processes modulated by KMU-11342 were identified by evaluating the 23 kinases exhibiting an inhibition of ≥80% in their activity. Protein–protein interaction (PPI) network analysis of the 23 highly inhibited kinases revealed that 19 kinases formed a significantly interconnected network (42 edges; *p* < 0.001) (Figure 1b), suggesting that these kinases function as a tightly linked regulatory module involved in shared biological processes. KEGG pathway analysis (Figure 1c) demonstrated significant enrichment in pathways associated with cell proliferation and survival, including the cell cycle (*p* < 0.001), MAPK signaling (*p* < 0.001), and PI3K–Akt signaling (*p* < 0.001). Gene Ontology (GO) analysis (Figure 1d) revealed significant enrichment of Biological Process terms such as peptidyl-serine phosphorylation (*p* < 0.001) and the G1/S transition of the mitotic cell cycle (*p* < 0.001), as well as Molecular Function terms, including protein serine kinase activity (*p* < 0.001) and cyclin-dependent protein kinase activity (*p* < 0.001). These findings suggest that KMU-11342 targets kinase networks involved in cell cycle regulation, proliferation, and survival-related signaling pathways.

### 2.3. Anti-Cancer Effects of KMU-11342 in Human Colon Cancer Cells

**Figure 2:**
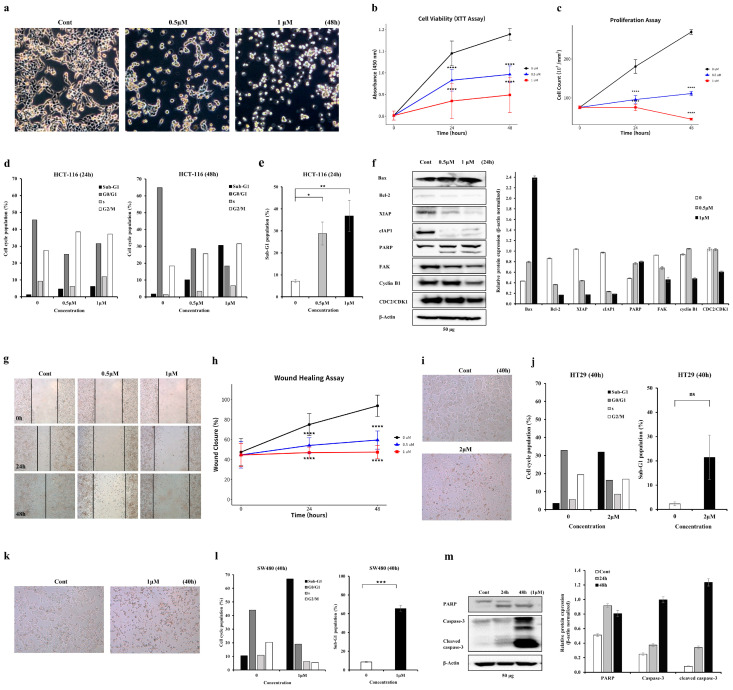
Anti-cancer effects of KMU-11342 in colorectal cancer cells. (**a**) Representative phase-contrast images of HCT116 cells treated with 0, 0.5, or 1 μM KMU-11342 for 48 h. (**b**) Cell viability assessed by XTT assay following treatment of HCT116 cells with 0, 0.5, or 1 μM KMU-11342 for 24 and 48 h. (**c**) Cell proliferation evaluated by cell counting following treatment of HCT116 cells with 0, 0.5, or 1 μM KMU-11342 for 24 and 48 h. (**d**) Flow cytometric analysis of cell cycle distribution in HCT116 cells after treatment with 0, 0.5, or 1 μM KMU-11342 for 24 and 48 h. (**e**) Quantification of the Sub-G1 population in HCT116 cells following treatment with 0, 0.5, or 1 μM KMU-11342 for 24 and 48 h. (**f**) Western blot analysis of apoptosis and cell cycle proteins in HCT116 cells following treatment with 0, 0.5, or 1 μM KMU-11342 for 24 and 48 h. (**g**) Wound healing assay in HCT116 cells treated with 0, 0.5, or 1 μM KMU-11342 at 0, 24, and 48 h. (**h**) Quantification of wound closure in HCT116 cells following treatment with 0, 0.5, or 1 μM KMU-11342 at 24 and 48 h. (**i**) Representative phase-contrast images of HT29 cells treated with 0 or 2 μM KMU-11342 for 40 h. (**j**) Flow cytometric analysis and quantification of the Sub-G1 population in HT29 cells following treatment with 0 or 2 μM KMU-11342 for 40 h. (**k**) Representative phase-contrast images of SW480 cells treated with 0 or 1 μM KMU-11342 for 40 h. (**l**) Flow cytometric analysis and quantification of the Sub-G1 population in SW480 cells following treatment with 0 or 1 μM KMU-11342 for 40 h. (**m**) Western blot analysis of cleaved PARP, caspase-3, and cleaved caspase-3 in SW480 cells following treatment with 1 μM KMU-11342 for 24 and 48 h. β-actin was used as a loading control. Densitometric quantification of protein expression was performed using ImageJ (version 1.52a) and normalized to β-actin. Data are represented as the mean ± SD. Statistical significance was determined using one-way ANOVA followed by Tukey’s post hoc test. * *p* < 0.05, ** *p* < 0.01, *** *p* < 0.001, **** *p* < 0.0001.

The anti-cancer effects of KMU-11342 were evaluated in HCT116 cells treated with KMU-11342 at 0, 0.5, and 1 μM for 24 and 48 h. Phase-contrast microscopy revealed a dose-dependent decrease in cell density, with apoptotic morphology observed at 1 μM (Figure 2a). Cell viability and proliferation assays demonstrated a significant dose- and time-dependent reduction, with the most pronounced effects at 1 μM (Figure 2b,c; *p* < 0.0001). Flow cytometry analysis at 24 and 48 h showed an increase in the Sub-G1 population and accumulation of cells in the G2/M phase (Figure 2d,e). Western blot analysis revealed apoptosis-related changes (Figure 2f). Bax expression and cleaved PARP levels increased, while the anti-apoptotic proteins Bcl-2, XIAP, and cIAP1 were downregulated. Key regulators of G2/M progression, including Cyclin B1 and CDK1, were also significantly reduced, consistent with cell cycle arrest. FAK expression was also decreased, suggesting its involvement in cell adhesion and survival pathways. These results indicate that KMU-11342 effectively inhibits HCT116 cell proliferation by inducing apoptosis and G2/M cell cycle arrest.

The effect of KMU-11342 on cell migration was assessed using a wound healing assay. HCT116 cells were treated with 0, 0.5, and 1 μM KMU-11342, and wound closure was assessed at 0, 24, and 48 h (Figure 2g). KMU-11342 inhibited cell migration in a dose-dependent manner, with reduced wound closure observed at higher concentrations (Figure 2h). Quantification of migration distance confirmed a significant reduction at both 24 and 48 h compared with the control (*p* < 0.0001). These findings suggest that KMU-11342 effectively inhibits HCT116 cell migration in a dose-dependent manner, consistent with an inhibitory effect on cancer cell motility.

Additional validation was performed in p53-mutant CRC cell lines, HT29 and SW480, to determine whether the anti-cancer effects of KMU-11342 were restricted to HCT116 cells. Phase-contrast microscopy revealed a reduction in cell density and the appearance of apoptotic morphology following KMU-11342 treatment in both cell lines (Figure 2i,k). Flow cytometric analysis demonstrated an increase in the Sub-G1 population in both cell lines after 40 h of treatment. Although the increase observed in HT29 cells did not reach statistical significance, a substantial increase in Sub-G1 cells was observed in SW480 cells (*p* < 0.001), indicating enhanced apoptosis (Figure 2j,l). Western blot analysis revealed elevated levels of cleaved PARP and cleaved caspase-3 at both 24 and 48 h following KMU-11342 treatment in SW480 cells (Figure 2m), further supporting the induction of apoptosis. These findings suggest that the anti-cancer effects of KMU-11342 are not restricted to HCT116 cells and also occur in p53-mutant CRC cell lines.

### 2.4. Anti-Cancer Effects of KMU-11342 on 3D Spheroid Growth and Organoid Viability

**Figure 3:**
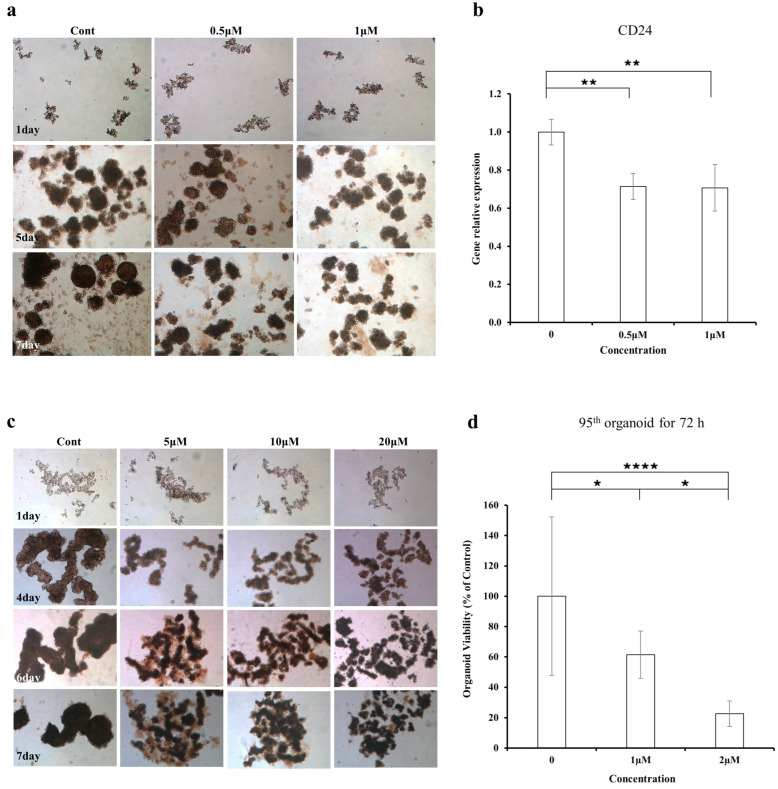
Anti-cancer effects of KMU-11342 on 3D spheroid formation and organoid viability. (**a**) Representative images of HCT116 spheroids treated with 0, 0.5, and 1 μM KMU-11342 at days 1, 5, and 7. (**b**) qPCR analysis of CD24 mRNA expression in HCT116 spheroids treated with 0, 0.5, and 1 μM KMU-11342, showing relative CD24 expression levels. (**c**) Representative images of HCT116 spheroids treated with 0, 5, 10, and 20 μM KMU-11342 during spheroid formation, observed at days 1, 4, 6, and 7. (**d**) Viability in patient-derived CRC organoids treated with 0, 1, and 2 μM KMU-11342. Data are represented as the mean ± SD. Statistical significance was determined using one-way ANOVA followed by Tukey’s post hoc test. * *p* < 0.05, ** *p* < 0.01, **** *p* < 0.0001.

The effect of KMU-11342 on 3D spheroid formation was evaluated using a sphere-formation assay under two treatment conditions. In the first condition, HCT116 spheroids were allowed to form for 4 days before treatment with 0, 0.5, and 1 μM of KMU-11342. Their 3D morphology was observed on days 1, 5, and 7 (Figure 3a). No substantial differences in spheroid morphology were observed at day 5 following KMU-11342 treatment. By day 7, treated spheroids appeared smaller than those in the control group, with reduced spheroid formation. To further characterize spheroid-associated features, CD24 expression was quantified using qPCR in spheroids harvested on day 7. CD24 expression was significantly reduced in KMU-11342-treated spheroids compared with the controls (*p* < 0.01) (Figure 3b), suggesting that KMU-11342 may affect spheroid-associated cellular features.

In the second condition, HCT116 spheroids were treated with 0, 5, 10, and 20 μM of KMU-11342 a day after seeding, and spheroid formation subsequently assessed (Figure 3c) to evaluate the effect of earlier treatment and higher concentrations. KMU-11342 suppressed spheroid formation in a dose-dependent manner, with near-complete suppression at 20 μM by day 7. These results demonstrate that KMU-11342 inhibits 3D spheroid formation in HCT116 cells in a dose- and time-dependent manner, and that reduction in CD24 expression may have an inhibitory effect on cancer stem-like properties.

The effect of KMU-11342 on patient-derived CRC organoid survival was evaluated using a viability assay. Organoids were allowed to form for 3 days and subsequently treated with 1 and 2 μM KMU-11342 for 48 h. KMU-11342 significantly reduced organoid viability in a dose-dependent manner (Figure 3d). Compared with the control group, the 1 μM-treated group showed a significant reduction in viability (*p* < 0.05), whereas the 2 μM-treated group exhibited a highly significant decrease (*p* < 0.0001), as well as a significant reduction compared with the 1 μM-treated group (*p* < 0.05). These findings demonstrate that KMU-11342 effectively suppresses CRC organoid survival, supporting its potential anti-cancer effects in a 3D organoid model.

### 2.5. Molecular Docking Simulation of KMU-11342 with CDK1 and GSK3β

Molecular docking simulations were performed to investigate the structural binding affinity between KMU-11342 and 12 *Homo sapiens*-derived protein structures, evaluated using Smina [12]. The in silico approach was conducted as previously described [13]. The binding sites for the two target proteins, CDK1 and GSK3β, were defined based on their respective ATP-binding pockets. This selection was consistent with the interaction patterns reported for SU6668, the motif compound of KMU-11342 [14], as both CDK1 and GSK3β harbor their active sites within this region [15,16]. Each docking simulation was performed in triplicate, and relative binding affinities were assessed based on average docking scores (Table S5).

Among the CDK1 structures, 6GU4 exhibited the strongest binding affinity (docking score of −10.3 kcal/mol; Table S3). For GSK3β, the structure 6HK7 showed the highest affinity (score of −9.7 kcal/mol; Table S4). In addition to docking scores, the orientation of KMU-11342 was further refined by referencing the binding conformation of SU6668 [14,17]. Specifically, docking poses in which the imidazole moiety of KMU-11342 was oriented toward the binding pocket were selected. Based on this criterion, CDK1 structures 6GU6 and 6GU7, and GSK3β structures 5K5N and 6HK4 were identified as the most appropriate. Although 6GU4 and 6HK7 showed stronger binding affinities, the differences were negligible (<0.5 kcal/mol). Thus, the selected structures were prioritized as they provided biologically representative conformations that preserved the indolin-2-one scaffold.

**Figure 4:**
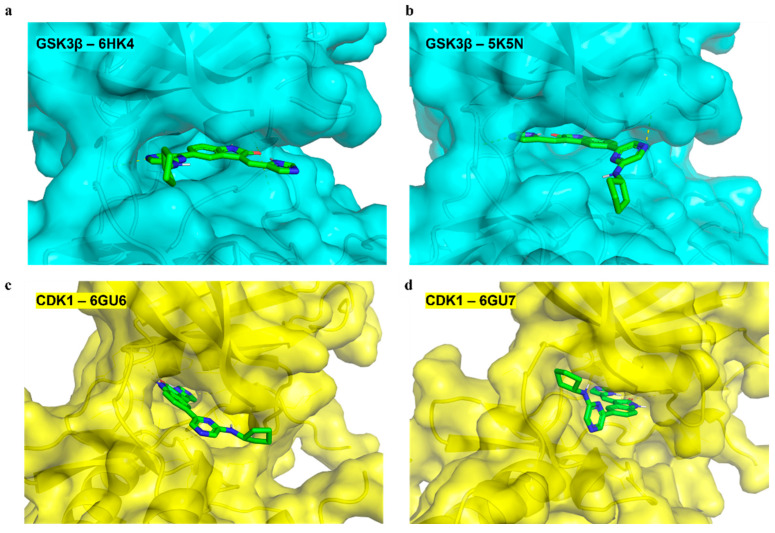
Surface representation of the predicted binding modes of KMU-11342 with GSK3β and CDK1. GSK3β and CDK1 are shown in cyan and yellow. Two potential binding conformations were identified for each kinase based on the reported structure of SU6668. (**a**) Surface view of KMU-11342 docked in GSK3β (PDB ID: 6HK4), showing placement within the hinge binding pocket. (**b**) Alternative conformation of KMU-11342 docked in GSK3β (PDB ID: 5K5N). (**c**) Surface view of KMU-11342 docked in CDK1 (PDB ID: 6GU6), illustrating its orientation within the catalytic pocket. (**d**) Alternative binding conformation of KMU-11342 in CDK1 (PDB ID: 6GU7). Residue contacts for each docking model are provided in Figure 5 and Figure 6, and full structural representations are shown in Figure S1.

Docking poses were examined as surface representations to visualize the binding disposition of KMU-11342 within each target, and two representative conformations were identified for each protein: the default orientation (Figure 4a,c) and its inverted form (Figure 4b,d). For GSK3β, the default pose positioned KMU-11342 at the hinge region (Figure 4a), consistent with the canonical ATP-competitive binding of kinase inhibitors. The alternative conformation retained occupancy of the same hinge-proximal region, indicating that both orientations converged on a similar binding site within GSK3β (Figure 4b). For CDK1, KMU-11342 adopted a distinct binding disposition, where the default pose directed the compound toward the catalytic/phosphate-binding region rather than the hinge region (Figure 4c), deviating from the canonical binding pattern observed for most ATP-competitive CDK1 inhibitors. The alternative conformation maintained engagement with the same catalytic/phosphate-binding area (Figure 4d), further supporting a preference of KMU-11342 for this non-canonical site in CDK1.

**Figure 5:**
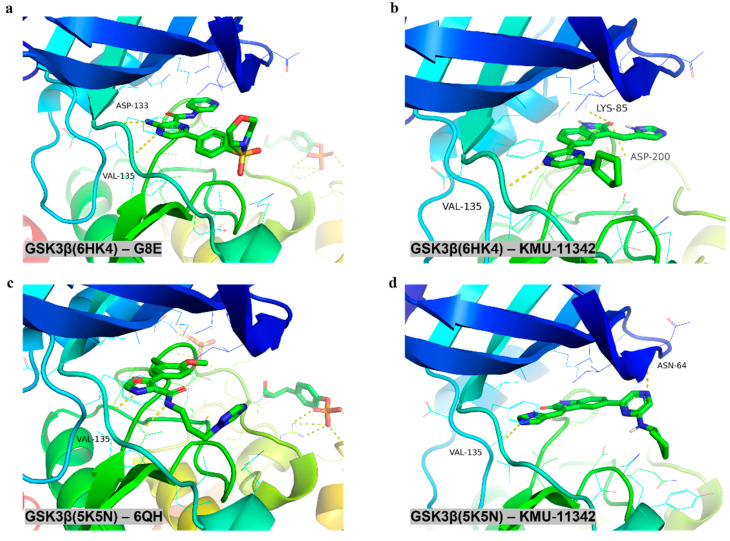
Residue contacts and putative hydrogen-bonding patterns of GSK3β inhibitors and KMU-11342. Key interacting residues are labeled, and yellow dashed lines indicate predicted hydrogen-bond interactions. (**a**) Binding mode of inhibitor G8E in GSK3β (PDB ID: 6HK4), showing its orientation relative to Asp133 and Val135. (**b**) Predicted binding mode of KMU-11342 in GSK3β (PDB ID: 6HK4), showing hydrogen-bond interactions with Val135 (3.37–3.49 Å), Asp200 (3.12 Å), and Lys85 (3.45 Å). (**c**) Binding of inhibitor 6QH in another GSK3β structure (PDB ID: 5K5N), interacting with Val135 (2.77_3.19 Å). (**d**) Predicted binding mode of KMU-11342 in GSK3β (PDB ID: 5K5N), showing hydrogen-bond interactions with Val135 (2.88 Å) and Asn64 (3.02–3.17 Å). A close-up view of the binding pocket is shown to highlight the ligand–protein interactions.

To validate the docking reliability, the predicted binding poses of KMU-11342 were compared with the co-crystallized reference ligands in two distinct GSK3β conformations (PDB IDs: 6HK4 and 5K5N). Figure 5a,c show the binding modes of the co-crystallized reference ligands in the two GSK3β conformations. In the default orientation, KMU-11342 occupied the ATP-binding site, forming key hydrogen bonds with Val135 (3.37_3.49 Å), Asp200 (3.12 Å), and Lys85 (3.45 Å) (Figure 5b). In the inverted form (Figure 5d), the interaction with Val135 (2.88 Å) was consistent with typical hinge region binding patterns.

**Figure 6:**
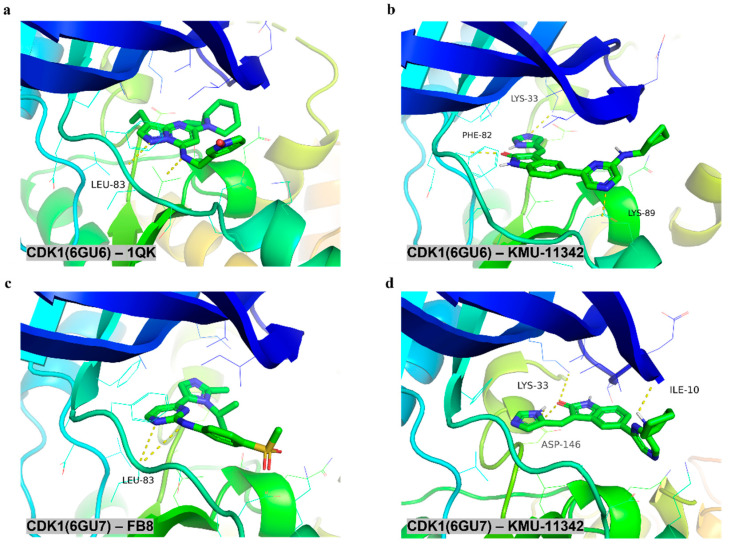
Hydrogen-bond distances and residue interactions between KMU-11342 and reference CDK1 inhibitors. Key interacting residues are labeled, and yellow dashed lines indicated predicted hydrogen-bond interactions. (**a**) Binding mode of Dinaciclib (1QK) in CDK1 (PDB ID: 6GU6), showing hydrogen-bond distances of 3.35–3.39 Å with Leu83. (**b**) KMU-11342 in the CDK1 structure (PDB ID: 6GU6), forming hydrogen bonds with Lys33 (2.86 Å) and Lys89 (3.24 Å), and a hydrophobic interaction with Phe82 (3.87 Å). (**c**) Binding of inhibitor FB8 in an alternative CDK1 structure (PDB ID: 6GU7), interacting with Leu83 (3.36 Å). (**d**) KMU-11342 in the CDK1 structure (PDB ID: 6GU7), interacting with Lys33 (3.39 Å), Asp146 (3.19 Å), and Ile10 via a hydrophobic interaction (3.55 Å). A close-up view of the binding pocket is shown to highlight the ligand-protein interactions.

Figure 6 shows the binding modes of the co-crystallized reference ligands in the two CDK1 conformations (Figure 6a,c). Leu83 in the hinge region of CDK1 is known to anchor the adenine moiety of ATP through key hydrogen bond interactions [18]. In the docking simulation, KMU-11342 exhibited a distinct binding profile, consistently lacking direct interactions with the Leu83 residue and instead forming stable interactions with Lys33, Lys89, and Asp146 (Figure 6b,d). Superimposition analysis revealed that KMU-11342 adopts a favorable conformation within the CDK1 catalytic pocket, closely mimicking the spatial arrangement of the crystallographic ligands. Overall, these docking results demonstrate favorable structural affinities and hydrogen-bonding interactions, supporting the potential of KMU-11342 to effectively target the ATP-binding region of GSK3β and the catalytic pocket of CDK1.

### 2.6. Prediction of the Biological Toxicity of KMU-11342

Using elEmBERT, we evaluated the toxicity of KMU-11342 across 12 distinct biological endpoints [19]. Non-toxicity probabilities (index 0) were extracted for each endpoint. KMU-11342 demonstrated favorable scores across all 12 toxicity endpoints, indicating an overall low toxicity profile. Although a relatively lower probability was observed for the NR-ER-LBD endpoint, this value remained within the non-toxic range (Table 2).

### 2.7. Effects of KMU-11342 on GSK3β and CDK1 Signaling

**Figure 7:**
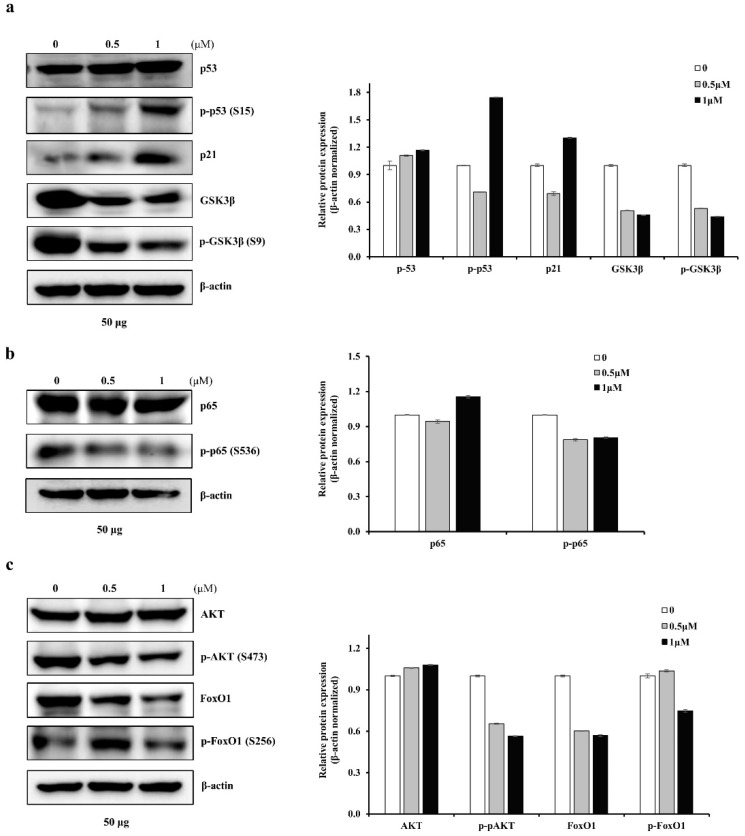
Effects of KMU-11342 on GSK3β and CDK1 signaling. (**a**) Western blot analysis of p53, phospho-p53 (S15), p21, GSK3β, and phospho-GSK3β (S9) in HCT116 cells treated with 0, 0.5, and 1 μM KMU-11342. (**b**) Western blot analysis of NF-κB p65 and phospho-p65 (S536) in HCT116 cells treated with 0, 0.5, and 1 μM KMU-11342. (**c**) Western blot analysis of AKT, phospho-AKT (S473), FoxO1, and phospho-FoxO1 (S256) in HCT116 cells treated with 0, 0.5, and 1 μM KMU-11342. β-actin was used as a loading control. Densitometric quantification of protein expression was performed using ImageJ and normalized to β-actin.

Kinase profiling and molecular docking analysis indicated that GSK3β and CDK1 are among the key kinases mediating the effects of KMU-11342. Both kinases regulate signaling pathways associated with cell cycle progression and survival [20,21,22]. The phosphorylation status of their key downstream signaling molecules was analyzed to determine whether KMU-11342 modulates the downstream signaling cascade. KMU-11342 increased the phosphorylation of p53 and the expression of p21 (Figure 7a), and reduced the phosphorylation of GSK3β, NF-κB p65, AKT, and FOXO1 (Figure 7b,c). These findings suggest that KMU-11342 modulates p53, NF-κB, and AKT–FOXO1 signaling pathways, which are critical regulators of cell cycle progression and survival, consistent with its anti-cancer effects.

## 3. Discussion

CRC is one of the most common and lethal malignancies worldwide. Recurrence, metastasis, and therapeutic resistance continue to hinder the effectiveness of current treatment strategies, underscoring the need for novel agents targeting alternative molecular pathways. KMU-11342, a multi-protein kinase inhibitor, was investigated in this study as a potential therapeutic candidate for CRC, with a focus on its underlying molecular mechanisms.

KMU-11342 exerted potent anti-cancer effects in HCT116 cells, suppressing proliferation and cell migration, while inducing G2/M cell cycle arrest and apoptotic cell death. These effects were further confirmed in 3D spheroid and patient-derived organoid models, supporting the relevance of these findings in more physiologically complex systems. These findings suggest that KMU-11342 may modulate key biological properties of CRC cells.

Kinase profiling revealed notable inhibitory activity against several kinases, including GSK3β, MST2, and CDK1/2/3. The kinases are essential regulators of cell cycle transitions, particularly the G1/S and G2/M checkpoints, and their inhibition is closely associated with G2/M arrest [18,20], consistent with our findings. GSK3β is a multifunctional kinase involved in apoptosis, differentiation, and cell fate, whose inhibition has been shown to promote apoptotic signaling in certain contexts [23]. MST2, a core component of the Hippo signaling pathway, contributes to stress-induced apoptosis and growth suppression [24]. Together, these kinases play diverse but complementary roles in controlling cancer cell proliferation and survival.

Molecular docking simulations were performed to investigate the predicted binding interactions of KMU-11342 with CDK1 and GSK3β. Protein structural flexibility presents a known challenge in computational modeling, as ligand binding and environmental conditions can alter protein conformation [25]. Nonetheless, KMU-11342 exhibited favorable docking scores with both CDK1 and GSK3β, suggesting strong and specific binding to both targets. These results should be interpreted as a structural hypothesis rather than definitive evidence of target engagement, as docking simulations assume a rigid protein conformation [15]. To address this limitation, molecular dynamics (MD) simulations are commonly employed as an essential in silico method to further assess structural stability and binding interactions over time, but cannot fully replace the importance of biological validation. Accordingly, the docking findings in this study are supported by parallel biological experiments confirming the functional activity of KMU-11342.

Pose selection was guided not only by docking score but also by preservation of the imidazole-oriented binding geometry characteristic of SU6668, the structural scaffold on which KMU-11342 was designed [14,17,26]. Given their high structural similarity, we considered that KMU-11342 within the ATP-binding pockets of CDK1 and GSK3β would adopt a binding orientation similar to the known conformation of SU6668 in its reference complex (e.g., TBK1). In CDK1, KMU-11342 consistently lacked direct interactions with Leu83, the canonical hinge residue, and instead occupied the catalytic/phosphate-binding region, engaging Lys33, Lys89, and Asp146. Considering that Lys33 is a well-established residue within the phosphate-binding pocket involved in coordinating the ATP phosphate groups [20], these structural features demonstrate that KMU-11342 targets the catalytic site via the phosphate-binding area, rather than interacting with the canonical hinge region. In contrast, the GSK3β poses supported a more conventional hinge-associated interaction, with KMU-11342 engaging Val135 across both evaluated structures. These target-dependent interaction patterns indicate that KMU-11342 does not operate through a single conserved binding mode, suggesting that its inhibitory consequences may differ across kinase contexts despite sharing a common scaffold. This structural flexibility highlights the capability of the indolin-2-one core to adapt to diverse catalytic environments, potentially explaining the multi-kinase inhibitory profile of KMU-11342.

The observed alterations in signaling pathways suggest that KMU-11342 exerts coordinated effects across multiple regulatory axes rather than acting on a single pathway. In the GSK3β-associated signaling axis, increased phosphorylation of p53 and the induction of p21 may reflect activation of tumor suppressor-mediated cell cycle control, consistent with previous studies demonstrating that GSK3β regulates p53-dependent transcriptional activity, including the expression of p21 [27]. In parallel, the reduction in phosphorylated NF-κB p65 suggests attenuation of NF-κB-dependent survival signaling, which is supported by prior evidence demonstrating that GSK3β contributes to NF-κB signaling through regulation of p65 phosphorylation and activation, and that inhibition of this pathway can promote apoptotic responses in cancer cells [28]. For the CDK1-associated signaling axis, the concomitant reduction in AKT and FOXO1 phosphorylation suggests that KMU-11342 may attenuate an AKT–FOXO1 survival and growth-regulatory axis in CRC cells. This finding is consistent with studies using genetic inactivation of AKT1 and AKT2 in human CRC cells, demonstrating that AKT-driven growth regulation is closely associated with FOXO1/FOXO3A phosphorylation [29]. CDK1 has been shown to directly phosphorylate FOXO1 and suppress its transcriptional activity, consequently promoting cell proliferation and survival [16]. Reduced phosphorylation of FOXO1 may reflect restoration of FOXO-related tumor-suppressive activity, while decreased AKT phosphorylation suggests disruption of upstream survival signaling. These signaling alterations are mechanistically consistent with the observed phenotypic changes in CRC cells. Downregulation of Cyclin B1 and CDK1 supports the induction of G2/M cell cycle arrest, while the increased Bax/Bcl-2 ratio and reduced expression of anti-apoptotic proteins XIAP and cIAP1 suggest activation of apoptosis through mitochondrial and survival pathway modulation. Increased PARP cleavage further suggests apoptosis and the decreased FAK expression suggests suppression of cell adhesion and migration. Together, these findings further strengthen the hypothesis that KMU-11342 exerts anti-cancer effects through coordinated regulation of cell cycle progression, apoptosis, and cell survival mechanisms.

Importantly, the activity of KMU-11342 in 3D spheroid and patient-derived organoid models extends its observed effects beyond conventional monolayer cultures. This model reflects the architectural complexity and cellular heterogeneity of tumors compared to conventional two-dimensional (2D) cultures [30,31,32], and the effect of KMU-11342 on this platform suggests that its biological activity is preserved in more complex and physiologically relevant systems.

Despite these findings, several limitations should be considered when interpreting the results of this study. First, docking results should be interpreted as structural hypotheses rather than definitive evidence of target engagement or inhibitory mechanism. In particular, we did not perform direct experimental validation to confirm whether KMU-11342 engages CDK1 through the predicted non-canonical binding mode, or whether this binding pose directly accounts for its inhibitory activity. Additionally, our docking analyses were limited to CDK1 and GSK3β; whether KMU-11342 adopts analogous non-canonical or hinge-directed poses in other kinase targets remains an open question that warrants broader computational and experimental profiling. Accordingly, the proposed binding modes and their mechanistic implications should be regarded as provisional, pending validation through structural biology studies, expanded kinase profiling, and cell-based functional assays. Second, to evaluate the biological toxicity of KMU-11342, we employed the deep learning-based model elEmBERT, which has demonstrated excellent predictive performance for toxicity endpoints related to nuclear receptor signaling and cellular stress response pathways. However, these endpoints primarily reflect toxicity at the cellular level and do not account for higher-order toxicities, such as hepatotoxicity or cardiotoxicity, which are crucial for drug development. While the in silico predictions and in vitro experiments indicated a low probability of cellular toxicity and anti-cancer efficacy, comprehensive in vivo evaluations and clinical studies will be required to fully validate the drug-like safety profile and therapeutic potential of KMU-11342. Lastly, the study lacked in vivo validation. Although the anti-cancer effects of KMU-11342 were consistently observed in CRC cells, spheroids, and patient-derived organoids, these findings were derived exclusively from in vitro and ex vivo models. Future studies using CRC xenograft models will therefore be necessary to determine whether the anti-cancer effects observed in vitro and ex vivo can be reproduced in a physiological tumor environment. Such studies will also facilitate evaluation of tumor growth inhibition and overall therapeutic efficacy under in vivo conditions. Ultimately, further in vivo and clinical investigations will be required to fully establish the translational potential of KMU-11342 for CRC treatment.

## 4. Materials and Methods

### 4.1. Synthesis and Kinase Profiling of KMU-11342

KMU-11342 synthesis and kinase profiling were conducted as previously described by Baek et al. [33]. The purity (>95%) of the compound was determined using high-performance liquid chromatography, and its structure confirmed by mass spectrometry and ^1^H/^13^C nuclear magnetic resonance. The kinase inhibitor KMU-11342 underwent kinase profiling using the Focused Cancer 67 Panel at Eurofins Cerep S.A. (Celle-Lévescault, France). This panel targets key oncogenic kinases associated with cell proliferation, tumor survival, cell-cycle, and metastasis (Table 1). The compound was evaluated at a concentration of 1 µM.

### 4.2. Functional Enrichment and Protein–Protein Interaction Network Analysis

Gene Ontology (GO) and Kyoto Encyclopedia of Genes and Genomes (KEGG) pathway enrichment analyses were performed using the clusterProfiler R package (version 4.10.1, Bioconductor) [34] to explore biological functions associated with the selected gene set. GO analysis encompassed three categories: Biological Process, Cellular Component, and Molecular Function. Terms with *p* < 0.05 were considered statistically significant. For visualization, representative enrichment results were selected based on gene count and statistical significance; specifically, five GO terms per category and ten KEGG pathways were presented.

Protein–protein interaction (PPI) network analysis was performed using the STRING database (https://string-db.org/, accessed on 17 April 2026) to investigate molecular interactions among kinases showing ≥80% inhibitory activity [35]. The resulting network was imported into Cytoscape (version 3.10.3) for visualization and analysis of network connectivity and functional relationships [36].

### 4.3. Protein Preparation and Molecular Docking Simulation

The target protein structures for molecular docking simulations were obtained from the RCSB Protein Data Bank (https://www.rcsb.org, accessed on 8 June 2026) and the PDBBind database (https://www.pdbbind.org.cn/, accessed on 8 June 2026), along with their co-crystallized ligands. For CDK1 and GSK3β, five and eight human-derived protein structures were selected, respectively. The SMILES notation of compound KMU-11342 was converted into a three-dimensional (3D), charge-assigned structure in PDBQT format using the Open Babel library [37], with the ‘mmff94s’ force field applied under pH 7.4 conditions to generate the 3D conformation. For docking, only the protein portion of each complex was isolated and converted into PDBQT format. The position and size of the binding pocket were determined by calculating the centroid and dimensions of the co-crystallized ligand within each protein-ligand complex. Molecular docking was performed using Smina (smina.osx.12), a fork of AutoDock Vina (AutoDock Vina 1.1.2), by placing KMU-11342 into the identified binding pockets to generate possible binding poses and corresponding binding scores. To ensure consistency, all docking simulations were performed in triplicate. All docking poses and molecular interactions were rendered using PyMOL (version 2.6, Schrödinger, LLC, New York, NY, USA). All interaction distances were calculated using PLIP (Protein–Ligand Interaction Profiler), Biotechnology Center Tu Dresden (BIOTEC), TUD Dresden University of Technology, Dresden, Germany [38].

### 4.4. Prediction of Biological Toxicity Using Artificial Intelligence

To evaluate the biological toxicity of KMU-11342, we employed elEmBERT [39], a large language model (LLM)-based deep learning framework that predicts compound toxicity from SMILES representations by incorporating molecular structure and atomic pair distributions. elEmBERT has demonstrated strong performance on the Tox21 benchmark dataset [40]. elEmBERT is available in two versions, V0 and V1. This study employed V1, which enhances performance by tokenizing interatomic distance distributions in conjunction with molecular features. Briefly, the model analyzes SMILES strings element-wise, performs pairwise atom distance distribution analysis, and classifies elements into subcategories for tokenization, increasing the number of learnable parameters. The tokenized inputs are then processed through embedding layers, multi-headed self-attention, and feed-forward layers to generate final outputs. All parameters were kept at their default settings, and training and evaluation were performed for 128 epochs with a batch size of 32. The final predictions were computed as 2D outputs via a SoftMax function.

A total of 12 toxicity endpoints were evaluated using the elEmBERT model. The output values range between 0 and 1, where values closer to 0 indicate higher toxicity, and values approaching 1 indicate lower toxicity. Conventionally, a threshold of 0.5 is used to distinguish between toxic and non-toxic predictions. The 12 endpoints assessed were: Androgen Receptor (NR-AR), Androgen Receptor Ligand Binding Domain (NR-AR-LBD), Aryl Hydrocarbon Receptor (NR-AhR), Aromatase (NR-Aromatase), Estrogen Receptor (NR-ER), Estrogen Receptor Ligand Binding Domain (NR-ER-LBD), Peroxisome Proliferator-Activated Receptor Gamma (NR-PPAR-γ), Antioxidant Response Element (SR-ARE), ATPase Family AAA Domain-Containing Protein 5 (SR-ATAD5), Heat Shock Element (SR-HSE), Mitochondrial Membrane Potential (SR-MMP), and p53 Signaling (SR-p53). Here, “NR” refers to toxicity related to nuclear receptor signaling pathways, and “SR” denotes to toxicity associated with cellular stress response pathways.

### 4.5. Cell Lines and Culture

HCT116 human colorectal carcinoma cells (p53 wild-type) obtained from the American Type Culture Collection (ATCC, Rockville, MD, USA) were maintained in an RPMI 1640 medium (Welgene Inc., Gyeongsan, Republic of Korea) supplemented with 10% heat-inactivated fetal bovine serum (FBS; Welgene Inc., Gyeongsan, Republic of Korea), 2 mM L-glutamine, 100 µg/mL streptomycin, and 100 µg/mL penicillin. Conditions within the incubator were regulated at 95% humidity and 37 °C, with a constant 5% CO_2_.

### 4.6. Cell Proliferation and Cell Counting Assay

HCT116 cell proliferation and counting assay were assessed using the XTT assay and direct cell counting with a hemocytometer. For the XTT assay (WEGENE), cells (20,000 per well) were seeded in a 96-well plate, allowed to adhere for 12 h, and then treated for an additional 48 h. The medium was discarded, 20 μL of XTT reagent was added to each well, and cells were incubated at 37 °C for 3 h. Absorbance was measured at 450 nm. For the cell counting assay, HCT116 cells were seeded at 0.6 × 10^6^ cells per well in 6-well plates and treated as indicated. After the designated incubation period, cells were trypsinized and resuspended in fresh medium. Viable cells were counted using a hemocytometer across four quadrants per well, with three replicates per sample.

### 4.7. Flow Cytometric Analysis

Approximately 0.3 × 10^6^ cells were suspended in 100 µL of phosphate-buffered saline (PBS), and 200 µL of 95% ethanol was added while vortexing. Cells were incubated at 4 °C for 1 h, washed with PBS, and resuspended in 250 µL of 1.12% sodium citrate buffer (pH 8.4) containing 12.5 µg RNase, followed by incubation at 37 °C for 30 min. Cellular DNA was then stained by adding 250 µL propidium iodide (50 µg/mL) for 30 min at 20–25 °C. Stained cells were analyzed using a FACScan flow cytometer (Becton Dickinson and Co., San Jose, CA, USA) to determine relative DNA content based on red fluorescence.

### 4.8. Western Blotting Analysis

Cellular lysates were prepared by suspending 0.5 × 10^6^ cells in 80 µL of RIPA buffer (20 mM HEPES and 0.5% Triton X-100, pH 7.6), followed by vortexing and extraction at 4 °C for 30 min. Protein concentration was determined using the BCA assay kit (Thermo Fisher Scientific, Waltham, MA, USA). Proteins were separated by SDS-PAGE and electrotransferred onto Immobilon-P membranes (Millipore Corp., Bedford, MA, USA). Specific proteins were detected using an ECL Western blotting kit (EMD Millipore, Darmstadt, Germany) according to the manufacturer’s instructions. Signal intensity was visualized with a Sapphire FL biomolecular imager (Azure Biosystems, Dublin, CA, USA; NFEC-2025-08-307766) and quantified using a Chemi Image System Fusion FX (Vilber Lourmat, Collégien, France).

The following antibodies were used: Anti-N-cadherin (sc-59987, 1:2000), anti-Snail (sc-3879, 1:500), anti-Bax (sc-493, 1:1000), anti-Bcl-2 (sc-7382, 1:500), anti-cIAP1 (sc-7943, 1:1000), FAK (sc-558, 1:700), Cyclin B1 (sc-752, 1:700), CDC2/CDK1 (sc-954, 1:1000), and p21 (sc-6246, 1:700) purchased from Santa Cruz Biotechnology (Dallas, TX, USA). The anti-β-actin (A5441, 1:10000) antibody and glutathione ethyl ester (GEE) were purchased from Sigma Chemical Co. (St. Louis, MO, USA). Anti-XIAP antibody (#6310762, 1:1000) was from BD Biosciences (San Jose, CA, USA). Anti-poly (ADP-ribose) polymerase (PARP) (#9542, 1:1000), anti-E-Cadherin (#14472, 1:2000), p-NF-κB p65 (#3033, 1:1000), NF-κB p65 (#4764, 1:1000), p-Akt (#8556, 1:1000), Akt (#9272, 1:1000), p-FoxO1 (#9461, 1:1000), FoxO1 (#2880, 1:1000), p-GSK3β (#9336, 1:700), GSK3β (#9332, 1:700), p-p53 (#9286, 1:1000), p53 (#2524, 1:1000), and anti-Vimentin (#5741, 1:1000) were purchased from Cell Signaling Technology (Danvers, MA, USA). Anti-rabbit IgG- horseradish peroxidase (HRP) and anti-mouse IgG-HRP secondary antibodies were purchased from Santa Cruz Biotechnology (Santa Cruz, CA, USA).

### 4.9. Wound Healing Assay

HCT116 human colorectal carcinoma cells were seeded in RPMI-1640 medium supplemented with 10% FBS for 24 h. Scratch wounds were then created using sterile pipette tips, and wound closure was monitored at 0, 24, and 48 h post-scratching using phase-contrast microscopy. Image analysis software (Image J, version 1.52a) was employed to quantify the extent of wound closure.

### 4.10. Spheroid Cultures

HCT116 spheroids were generated using the ultra-low attachment (ULA) plate method. Briefly, 10,000 cells per well were seeded into 24-well plates (Corning) and cultured in RPMI 1640 medium supplemented with 10% FBS, 2 mM L-glutamine, 100 µg/mL streptomycin, and 100 µg/mL penicillin at 37 °C in 5% CO_2_. KMU-11342 was administered at concentrations of 0, 0.5, 1, 5, 10, and 20 μM depending on the experimental condition. Spheroids were cultured for 7 days and imaged during the culture period.

### 4.11. RNA Isolation and Quantitative Real-Time PCR

Total RNA was isolated using TRIzol reagent (Invitrogen, San Diego, CA, USA) according to the manufacturer’s protocol. HCT116 spheroids were cultured and treated as defined in Section 4.10, following which they were harvested and RNA purity and concentration were assessed using a NanoDrop spectrophotometer (Thermo Fisher Scientific, Waltham, MA, USA). For cDNA synthesis, 1 µg of total RNA was reverse-transcribed using SuperScript II reverse transcriptase (Thermo Fisher Scientific, Waltham, MA, USA). qPCR was performed using SYBR Green Master Mix (Applied Biosystems, Thermo Fisher Scientific, Waltham, MA, USA) on a QuantStudio™ 3 Real-Time PCR System (Applied Biosystems). The expression of CD24 was analyzed using the following primers: Forward: 5′-CTCCTACCCACGCAGATTTATTC-3′; Reverse: 5′-AGAGTGAGACCACGAAGAGAC-3′. Results were normalized to GAPDH (Forward: 5′-TTGGTATCGTGGAAGGACT-3′; Reverse: 5′-GGATGATGTTCTGGAGAGC-3′) using the ΔΔCt method.

### 4.12. Organoid Culture and Viability Assay

Patient-derived organoids were established using fresh tumor tissue samples obtained from a patient diagnosed with CRC at Keimyung University Dongsan Hospital (approved by the Keimyung University Dongsan Hospital Institutional Review Board; IRB Nos. 2019-09-021 and 2019-12-047). The tumor tissues were dissociated using Gentle Cell Dissociation Reagent, and the isolated cells were resuspended in DMEM/F12 (STEMCELL Technologies, Vancouver, BC, Canada). The cell suspension was mixed with growth factor-reduced Matrigel (Corning, NY, USA) at a 1:1 ratio. Organoids were cultured in IntestiCult™ Organoid Growth Medium (STEMCELL Technologies, Vancouver, BC, Canada) at 37 °C in 5% CO_2_, supplemented with Y-27632, a Rho-kinase inhibitor, for the first 2–3 days. Medium was changed every 2–3 days, and organoids were passaged weekly using a 70 μm cell strainer. Passage-four organoids, cultured for at least 14 days, were used for all experiments. For the viability assay, organoids were seeded in 96-well plates (1000 cells/well) and allowed to form for 3 days before treatment with 1 and 2 μM KMU-11342 for 48 h. Viability was assessed using luminescence measurement following organoid lysis with CellTiter-Glo^®^ 3D Cell Viability Assay (Promega, Madison, WI, USA) 3D reagent. Luminescence was recorded following 25 min of stabilization at room temperature (20–25 °C) in the dark. All experiments were performed in triplicate.

### 4.13. Statistical Analysis

All statistical analyses were performed using R software (version 4.3.3, R Foundation for Statistical Computing, Vienna, Austria). For comparisons among multiple groups, one-way analysis of variance (ANOVA) was performed, followed by Tukey’s post hoc test for multiple comparisons. For pairwise comparisons, Student’s *t*-test was applied where applicable. For GO and KEGG pathway enrichment analyses, *p*-values were calculated using Fisher’s exact test based on the hypergeometric distribution. Data are presented as mean ± standard deviation (SD). Statistical significance was set at *p* < 0.05.

## 5. Conclusions

KMU-11342 exhibited potent anti-cancer activity in CRC models by suppressing cell proliferation and migration while inducing G2/M cell cycle arrest and apoptosis. These anti-cancer effects were consistently observed in both conventional 2D cultures and more physiologically relevant 3D models, including spheroids and patient-derived organoids. Kinase activity profiling and molecular docking analyses identified GSK3β and CDK1 as potential mediators of the observed biological effects, and the associated alterations in p53/NF-κB and AKT/FOXO1 signaling pathways further support the involvement of multiple signaling pathways in the anti-cancer effects of KMU-11342. Collectively, these findings demonstrate the anti-cancer activity of KMU-11342 in CRC models, supporting further investigation of its therapeutic potential. However, further in vivo studies and additional mechanistic validations will be required to fully establish its clinical applicability and molecular mode of action.

## Figures and Tables

**Table 1:** Kinase activity of 67 protein kinases following treatment with 1 µM KMU-11342.

Kinase	Activity (% Control)	Function
GSK3β	−3	Apoptosis resistance
MST2	−2	Tumor suppressor
CDK1/Cyclin B	−1	Cell cycle check point control
Abl	0	Oncogenic signalization
CDK2/Cyclin A	0	Cell cycle check point control
CDK5/p35	1	Cell cycle check point control
CDK2/Cyclin E	2	Cell cycle check point control
CDK3/Cyclin E	5	Cell cycle check point control
CDK6/Cyclin D3	5	Cell cycle check point control/Major therapy in cancer
Rsk1	5	Cellular proliferation and survival
PKD2	6	DAG/Ca^2+^ signaling. Oncogene or tumor suppressor depending on the isoform
PAR-1Bα	8	Cell polarity
PRK2	8	Cell cycle progression and migration
c-RAF	10	Cell proliferation
Rsk1 (rat)	10	Cell proliferation
PDGFRb	11	Frequent mutations/Amplifications in solid cancers
Rsk3	11	Cell proliferation
PKCμ	12	DAG/Ca^2+^ signaling. Oncogene or tumor suppressor depending on the isoform
p70S6K	15	Cell growth and proliferation
PDK1	15	Apoptosis resistance
Rsk2	16	Cell proliferation
CHK2	19	DNA damage response and checkpoint regulation
Fms	19	Frequent mutations/Amplifications in solid cancers
MAPK2	22	Cell proliferation
Abl (T315I)	31	Oncogenic signalization
MEK1	32	Cell proliferation
CDK7/Cyclin H/MAT1	33	Cell cycle check point control
CaMKIIβ	36	Ca^2+^/Calmodulin signaling
PKCθ	39	DAG/Ca^2+^ signaling. Oncogene or tumor suppressor depending on the isoform
CK2	43	Cell cycle progression and apoptosis
EphB4	43	Cell migration and angiogenesis
Aurora-A	48	Mitosis regulation, Chromosomic instability
FGFR3	49	Frequent mutations/Amplifications in solid cancers
SGK	49	Apoptosis resistance
PAK2	54	Cell survival, proliferation and migration
PKCδ	58	DAG/Ca^2+^ signaling. Oncogene or tumor suppressor depending on the isoform
EGFR	59	Frequent mutations/Amplifications in solid cancers
Tie2	61	Frequent mutations/Amplifications in solid cancers
Arg	62	Oncogenic signalization
PKBα	70	Apoptosis resistance
PKCε	70	DAG/Ca^2+^ signaling. Oncogene or tumor suppressor depending on the isoform
EphB2	72	Cancer migration invasion and angiogenesis
PDGFRa	73	Frequent mutations/Amplifications in solid cancers
PKCα	73	DAG/Ca^2+^ signaling. Oncogene or tumor suppressor depending on the isoform
CHK1	75	Control of DNA damage and check point regulation
CK1δ	76	DNA repair, apoptosis and cell cycle regulation
IGF-1R	76	Frequent mutations/Amplifications in solid cancers
PI3Kinase (p110α/p85α)	76	Apoptosis resistance
PI3 Kinase (p110α(E542K)/p85α)	78	Apoptosis resistance
Met	79	Frequent mutations/Amplifications in solid cancers
CaMKIV	80	Ca^2+^/calmodulin signaling and transcriptional regulation
CSK	82	Migration, invasion and metastasis
PI3 Kinase (p110α(H1047R)/p85α)	82	Apoptosis resistance
PKBγ	82	Apoptosis resistance
PKCγ	83	DAG/Ca^2+^ signaling. Oncogene or tumor suppressor depending on the isoform
NEK2	84	Mitosis regulation, chromosomic instability
ATM	89	Control of DNA damage and check point regulation
PI3 Kinase (p110α(E545K)/p85α)	90	Apoptosis resistance
PKCβII	93	DAG/Ca^2+^ signaling. Oncogene or tumor suppressor depending on the isoform
PKCι	93	DAG/Ca^2+^ signaling. Oncogene or tumor suppressor depending on the isoform
ATR/ATRIP	95	Control of DNA damage and check point regulation, important target in therapeutics
PKBβ	101	Apoptosis resistance
Ros	110	Fusion oncogene
PKCη	111	DAG/Ca^2+^ signaling. Oncogene or tumor suppressor depending on the isoform
CK1	113	Positive regulation of cell migration
PKCζ	115	DAG/Ca^2+^ signaling. Oncogene or tumor suppressor depending on the isoform
PRAK	129	Tumor suppressor and cytoskeletal dynamics

**Table 2:** Biological toxicity prediction of KMU-11342 using a deep learning model. Predicted toxicity profile of KMU-11342 across 12 Tox21 endpoints, including nuclear receptor (NR) and stress response (SR) pathways, using the elEmBERT2 deep learning model.

	KMU-11342
NR-AR	0.99562100
NR-AR-LBD	0.98555850
NR-AHR	0.99872040
NR-Aromatase	0.99987760
NR-ER	0.94749510
NR-ER-LBD	0.87142910
NR-PPAR-γ	0.99981980
SR-ARE	0.99788034
SR-ATAD5	0.99928420
SR-HSE	0.98738146
SR-MMP	0.99832980
SR-p53	0.99965465

## Data Availability

The original contributions presented in this study are included in the article and supplementary material. Further inquiries can be directed to the corresponding authors.

## Supplementary Materials

The following supporting information can be downloaded at: https://www.mdpi.com/article/10.3390/ph19070985/s1, Figure S1: Full structural image of the GSK3β-KMU-11342 and CDK1-KMU-11342 docking complexes; Table S1: GO_results; Table S2: KEGG_results; Table S3: Docking score profiles for CDK1; Table S4. Docking score profiles for GSK3β; Table S5. Docking score of the complexes in Figure 1.
