# Supplementary material for: Evaluation of the Anti-Cancer Effects of KMU-11342 in In Vitro and Ex Vivo Models of Colorectal Cancer
Source: Pharmaceuticals (Basel). 2026 Jun 25;19(7):985. doi: 10.3390/ph19070985 (PMC13415177; doi:10.3390/ph19070985)
Supplement: Supplementary file 1 [file pharmaceuticals-19-00985-s001.zip › Supplementary Tables S3,S4,S5 and Supplementary Figure S1.pdf]

Table S3. Docking score profiles for CDK1.

All scores are reported in kcal/mol. Docking affinity values from three independent docking simulations of KMU-11342 against CDK1, together with the average binding affinity.

	PDB code	Ligand Name	1st docking	2nd docking	3rd docking	average
1	6GU2	F9Z	-8.3	-8.5	-8.4	-8.4
2	6GU3	FB8	-10.1	-10.3	-10.2	-10.2
3	6GU4	FC8	-10	-10.1	-10	-10.03
4	**6GU6**	1QK	-8.8	-8.8	-8.8	-8.8
5	**6GU7**	FB8	-8.1	-8	-7.9	-8

Table S4. Docking score profiles for GSK3β.

All scores are reported in kcal/mol. Docking affinity values from three independent docking simulations of KMU-11342 against GSK3β, together with the average binding affinity.

	PDB code	Ligand Name	1st docking	2nd docking	3rd docking	average
1	2OW3	BIM	-8.9	-8.9	-8.9	-8.9
2	3Q3B	55E	-7.6	-8	-7.6	-7.73
3	5F94	3UO	-8.6	-8.9	-8.6	-8.7
4	5F95	3UP	-8.5	-9.2	-8.5	-8.73
5	**5K5N**	6QH	-9.6	-9.4	-9.6	-9.53
6	6HK7	G8N	-9.9	-9.3	-9.9	-9.7
7	**6HK4**	G8E	-9.4	-9.4	-9.4	-9.4
8	6HK3	G8B	-9.1	-9.2	-9.1	-9.13

Table S5. Docking score of the selected complexes.

Docking affinity values of the representative CDK1 and GSK3β structures selected for final structural and interaction analysis.

	PDB Code	Docking score
1	6GU6	-7.5
2	6GU7	-8.1
3	6HK4	-9.4
4	5K5N	-9.4

Figure S1. Full structural image of the GSK3β-KMU-11342 and CDK1-KMU-11342 docking complexes.

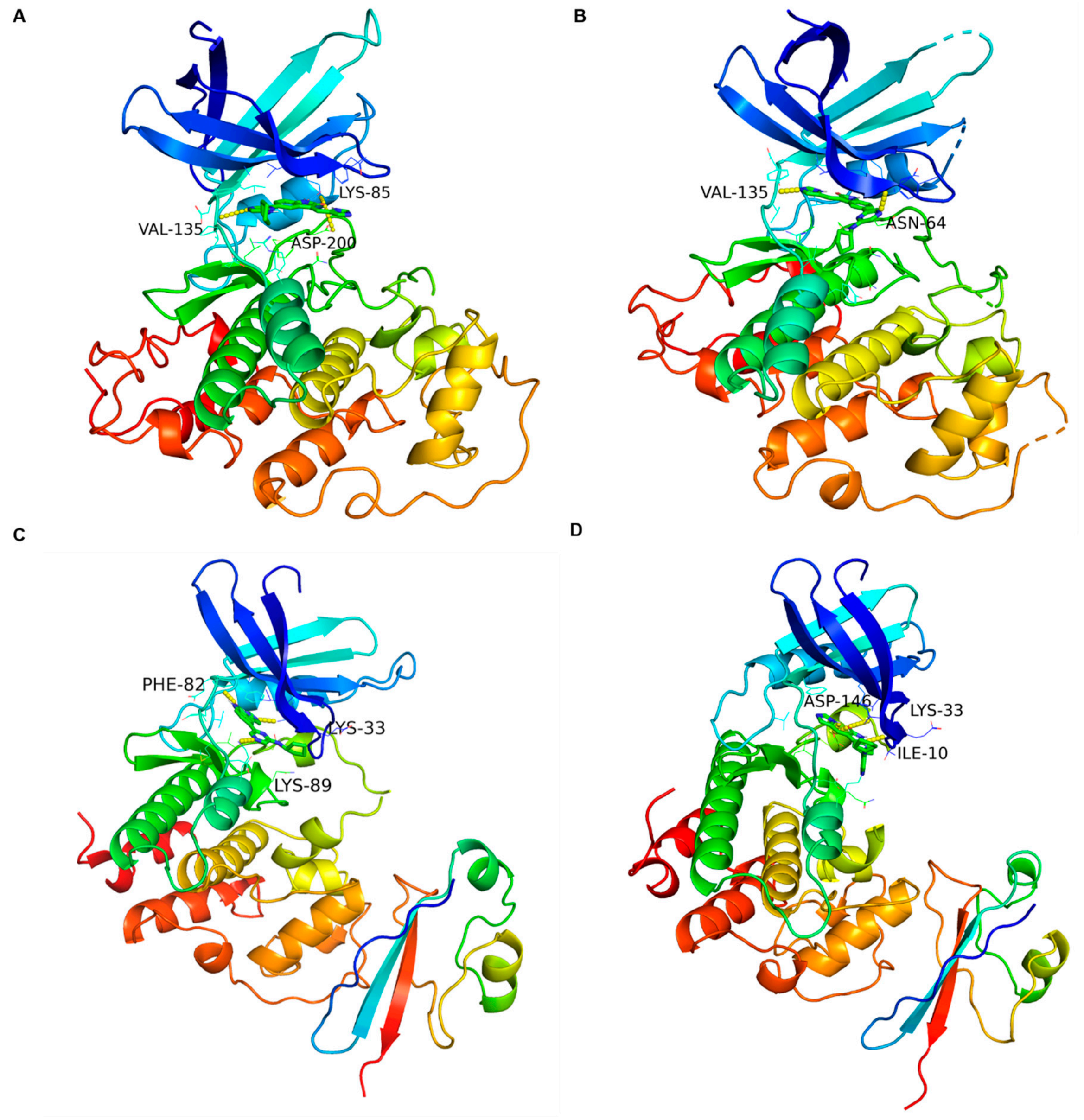

(A) Full structure of GSK3β with KMU-11342 (PDB ID: 6hk4). (B) Another full structure of GSK3β with KMU-11342 (PDB ID: 5k5n). (C) Full structure of CDK1 with KMU-11342(PDB ID: 6GU6). (D) Another full structure of CDK1 with KMU-11342 PDB ID: 6GU7).
